# A new approach to radon temporal correction factor based on active environmental monitoring devices

**DOI:** 10.1038/s41598-021-88904-2

**Published:** 2021-05-11

**Authors:** T. Dicu, B. D. Burghele, M. Botoş, A. Cucoș, G. Dobrei, Ș. Florică, Ș. Grecu, A. Lupulescu, I. Pap, K. Szacsvai, A. Țenter, C. Sainz

**Affiliations:** 1 “Constantin Cosma” Radon Laboratory (LiRaCC), Faculty of Environmental Science and Engineering, “Babeş-Bolyai” University, Str. Fântânele 30, Cluj-Napoca, Romania; 2 Faculty of Civil Engineering, Technical University of Cluj-Napoca, Str. C. Daicoviciu 15, Cluj-Napoca, Romania; 3 Faculty of Biology and Geology, Department of Geology, “Babeş-Bolyai” University, Str. M. Kogalniceanu 1, Cluj-Napoca, Romania; 4 Department of Medical Physics, Faculty of Medicine, University of Cantabria, c/ Herrera Oria s/n, 39011 Santander, Spain

**Keywords:** Lung cancer, Pollution remediation, Atmospheric science, Natural hazards

## Abstract

The present study aims to identify novel means of increasing the accuracy of the estimated annual indoor radon concentration based on the application of temporal correction factors to short-term radon measurements. The necessity of accurate and more reliable temporal correction factors is in high demand, in the present age of speed. In this sense, radon measurements were continuously carried out, using a newly developed smart device accompanied by CR-39 detectors, for one full year, in 71 residential buildings located in 5 Romanian cities. The coefficient of variation for the temporal correction factors calculated for combinations between the start month and the duration of the measurement presented a low value (less than 10%) for measurements longer than 7 months, while a variability close to 20% can be reached by measurements of up to 4 months. Results obtained by generalized estimating equations indicate that average temporal correction factors are positively associated with CO_2_ ratio, as well as the interaction between this parameter and the month in which the measurement took place. The impact of the indoor-outdoor temperature differences was statistically insignificant. The obtained results could represent a reference point in the elaboration of new strategies for calculating the temporal correction factors and, consequently, the reduction of the uncertainties related to the estimation of the annual indoor radon concentration.

## Introduction

In the last decades, the general public has become more and more interested in the influence that environmental issues have on their lifestyle and health. At present, numerous studies are aimed towards identifying the pollutants of the environment we live in and where we conduct our daily activities. Indoor pollutants can have both natural and artificial origins. In terms of natural pollutants special attention is paid to natural radioactivity. Radon gas is one of the most studied indoor pollutants at present, in term of natural radioactivity. The production of radon (^222^Rn) depends on the activity concentrations of radium (^226^Ra) in the Earth’s crust, in soil, rocks or building materials. Radon concentration levels are strongly affected by atmospheric influences such as temperature, pressure and humidity. The exhalation of radon from soil has been positively correlated with moisture content, temperature and negatively with pressure, so that these factors must be considered in the determination of exhalation rates in environmental measurements^1^. Although it can be found outdoors as well as indoors, due to dispersion and dilution, it can hardly accumulate in relevant concentrations in open spaces, as opposed to indoor where ventilation is limited. Being water soluble and chemically inert, radon can easily penetrate many common building materials, ultimately accumulating inside buildings. Numerous studies pointed out that prolonged exposure to high concentrations of radon indoors could have unwanted consequences for human health^2,3^. Scientists warn that radon exposure is the second leading cause of lung cancer, after smoking^4–6^. Cancer of the lung is known to have the highest rate of mortality due to this disease in the world^7^. Screening measurements, already carried out throughout Romania^8–10^, pointed out that radon induced lung cancer is not uncommon in the country. Therefore, radon screening measurements are important for the safety and health-care of people, and should be a building block for sustainable development of territories.

The indoor radon concentration in the same environment presents both diurnal and seasonal variation, as a result of the changing of environmental factors (temperature, pressure, humidity), respectively of the behaviour of the building’s inhabitants. The assessment of the annual indoor radon concentration (AIRC) is most often based on short-term passive measurements, using track detectors^11–13^, which prompted the need to use certain factors (temporal correction factors—TCF) in order to correct the fluctuations mentioned above^14–17^. Various studies, however, also report significant variations in the temporal correction factors, from one building to another, within the same measurement interval^18,19^, especially during the hot season^19–22^. Applying a regional or national average value for the temporal correction factor will lead to under/over estimation of true indoor radon exposure^23–26^, which could have long-term repercussions on human health^3,27^. Therefore, it is recommended that either the passive detectors to be installed for a longer period of time, i.e. 6–12 months^28–31^, or to reduce the uncertainties associated with the temporal correction factor assessment by other means^32–36^. The increase of measurement period, however, is not often an option in the present age of speed, especially in countries where real estate transactions are conditioned by the existence of a radon measurement^37,38^.

In the recent years, there has been an increase in the number of devices capable of continuous, real-time monitoring of numerous indoor pollutants, including radon. This trend has been shaped both by the reduction and miniaturization of the sensors, respectively the increase of their accuracy, as well as a result of the increase of the awareness about the importance of the indoor air quality. A viable indirect path to reduce the uncertainties associated to TCF could be to monitor additional parameters (indoor temperature, relative humidity, CO_2_, etc.) which could provide details related to synergistic effects on the distribution of the temporal correction factors. In this way, an adjusted value according to the details provided by the additional measurements will be used for the correction factor in order to assess the AIRC. Along these lines, the error associated with the estimation of the AIRC will be reduced compared to the scenario in which a national average value for the TCF would be used.

In this regard, a longitudinal balanced study was conducted in order to identify and analyse the association between radon temporal correction factors and indoor CO_2_ concentration, as well as other investigated parameter (temperature and relative humidity). Active devices, named ICA system, developed by the members of LiRaCC were placed in 100 energy efficient residential buildings from Romania during one year with the purpose of acquiring the necessary information. In order to capture the changes in routine among home users, the ratio between the annual CO_2_ concentration and the average for each month was used. A positive relation between the TCF for radon concentration and the CO_2_ ratio is expected. An increased value for the latter would mean a below average presence of home users, corroborated with a good ventilation, which would create the perfect conditions for an increase for the TCF (low indoor radon concentration). On the other hand, an inverse relation between the temperature differences (indoor-outdoor) and the TCF is anticipated, taking into account the fact that the highest values ​​for TCF are specific to the hot season, in which situation the temperature difference between the two environments is the lowest.

The method of generalized estimating equations (GEE) was used to analyse the recorded data. As Salazar et al.^39^ pointed out, the GEE is an extension of generalized linear models (GLMs) to longitudinal data, where observations are no longer independent. The GEE method models the mean changes of the outcome over time, as well as the impact of the covariates on these changes^40,41^.

In the same time, an in situ comparison between the indoor radon concentrations obtained with ICA system and CR-39 track detectors was made in order to emphasize the accuracy offered by the new environmental monitoring devices compared to the “golden standard” for radon measurements—the passive method.

## Results

### An overview of indoor radon concentrations (IRC)

The median of the construction year for the selected houses was 1975, 33 (46%) being constructed during the communist period (1955–1989), while 25 (35%) were built after 2000.

The remaining houses (18%) were built between 1900–1954 and 1990–1999. About 92% of the houses have a form of thermal insulation applied on the outside walls, the main type of window being PVC insulating glass. In up to 80% of the houses a concrete slab is present at the house-soil interface, while in 34% (24) of cases a basement was identified under the investigated room.

Due to the log-normal distribution of radon measurements, confirmed by applying the Shapiro–Wilk test, the preferred central tendency indicator was the geometric mean rather than the arithmetic mean. The AIRC highlighted geometric means of 296 Bq/m^3^ for passive measurements and 266 Bq/m^3^ for active measurements, respectively. As can be seen in Table 1, the highest values for geometric means are specific to the winter season (GM_p_ = 515 Bq/m^3^, GM_a_ = 440 Bq/m^3^), while the lowest means correspond to the summer season (GM_p_ = 128 Bq/m^3^, GM_a_ = 120 Bq/m^3^). By applying the ANOVA test on repeated measures, radon concentrations change significantly from one season to another, regardless of the measurement method used. By applying Mann–Whitney test (M-W test) or Kruskal–Wallis test (K–W test) on the AIRC or IRC, regardless of the selected independent variable, represented by the building characteristics, no significant differences were registered between the samples. As computed with K-W test, a statistically significant difference was obtained between IRC for the spring and summer seasons according to the city in which the measurement took place. However, the unbalanced number of measurements between cities, must be taken into consideration. Descriptive statistics of IRC depending on the city and the season are represented in Table S1.

**Table 1:** The descriptive statistics of indoor radon concentration (Bq/m^3^) depending on the season and the measurement method in 71 retrofit houses.

Period of time	Measurement method	Min	Max	A.M	S.D	Med	M.A.D	G.M	G.S.D
Spring	p	64	711	313	143	286	101	277	1.69
	a	81	553	284	129	276	96	254	1.66
Summer	p	37	318	143	66	121	46	128	1.63
	a	36	305	135	63	120	43	120	1.61
Autumn	p	152	1108	460	199	419	120	420	1.53
	a	138	950	417	195	388	148	370	1.63
Winter	p	197	1654	586	324	493	169	515	1.65
	a	160	1183	490	230	430	133	440	1.60
Annual	p	116	601	317	114	314	85	296	1.46
	a	106	515	288	107	290	83	266	1.50

p—passive measurements using CR-39 detectors; a—active measurements using ICA system with TSRS2 radon sensor; A.M.—arithmetic mean; S.D.—standard deviation; Med.—Median; M.A.D.—Median Absolute Deviation; G.M.—geometric mean; G.S.D.—geometric standard deviation.

The ratios between winter and summer radon concentrations (r_w/s_) for the passive measurements showed a lognormal distribution with a geometric mean of 4.03 and the range of values between 1.34 and 17.72. In the case of the active method, the r_w/s_ values range between 1.38 and 12.04 with a geometric mean of 3.63. By applying the paired t-test, a statistically significant difference was obtained between the pairs of r_w/s_ calculated for the two radon measurement methods (t = 2.68, df = 70, *p* = 0.001). The range of values ​​is much narrower (0.48–3.95) in the case of ratios between autumn and spring IRC compared to r_w/s_, with an identical geometric mean of 1.45 for the two measurements methods. Paired t-test applied to passive and active measurements of r_a/s_ did not highlight statistically significant differences (t = 0.82, df = 70, *p* = 0.42).

**Figure 1:**
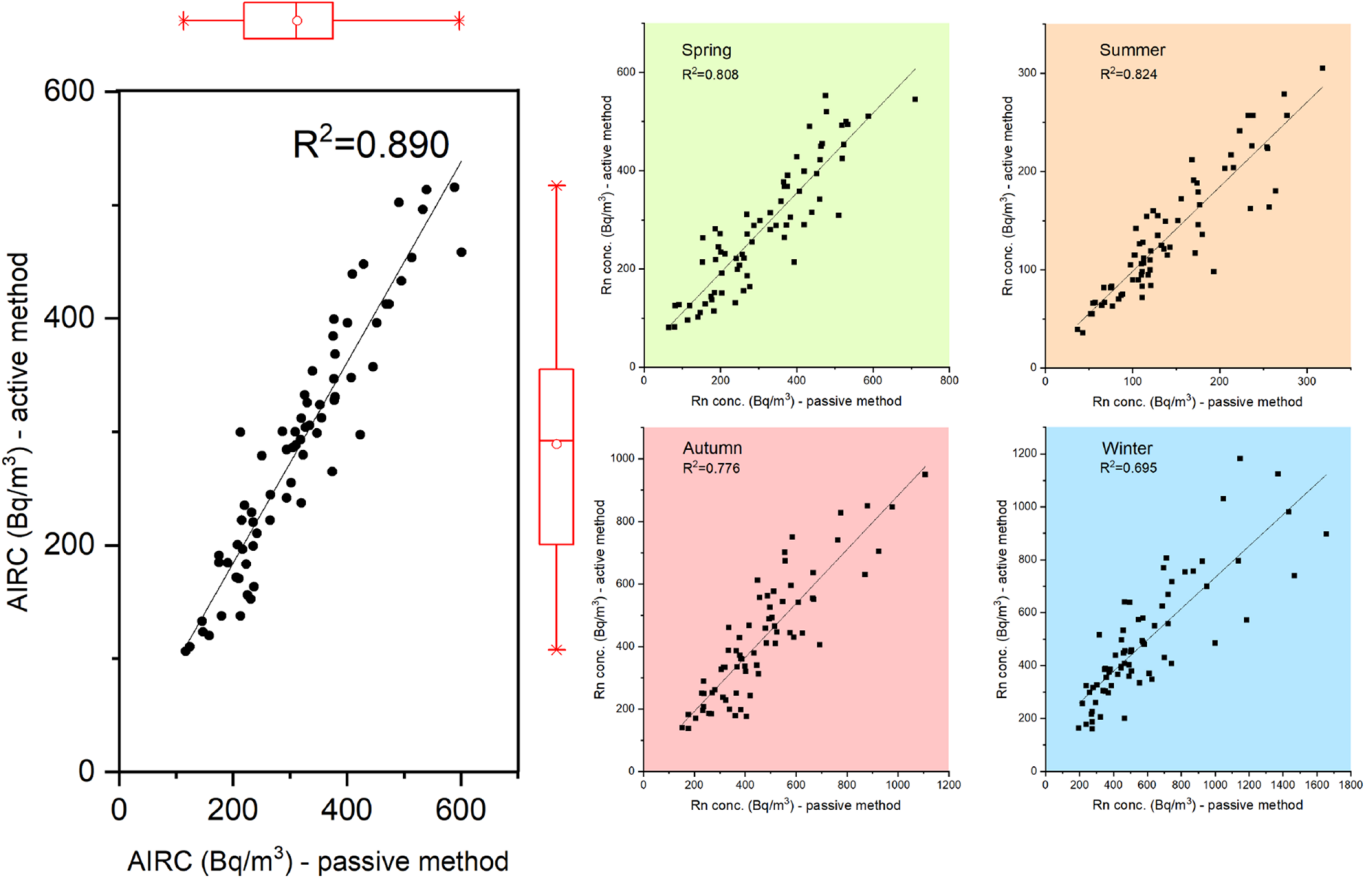
Scatterplots between passive and active methods (left side); the figure also shows the box-plots of data distribution. The same analysis is shown according to the measurement periods (right side). The figure was made using OriginPro 2019b software (www.originlab.com).

A strong linear correlation was found between the AIRCs obtained using the two methods, aspect confirmed by the Pearson correlation coefficient (r = 0.944, n = 71, *p* < 0.01) and shown in Fig. 1. At seasonal level, the correlation coefficients presented r values between 0.836 (winter) and 0.926 (summer). The slope of the regression line between the two methods in terms of AIRC was 0.884, with an intercept of 7.39. The Lin’s concordance correlation coefficient (r_C_ = 0.92) indicates a good agreement between the two methods. At seasonal level, the slopes were 0.592 for the winter season, 0.813 for the spring season and 0.862 for summer and autumn seasons, respectively. If the intersection was between 12.46 for the summer and 29.94 for the spring season, in the case of the winter season the intercept was up to 142. Following the regression analysis, it was obtained that the slope is statistically significant different from zero regardless of the season, while the intersection is significantly different from zero only for the winter season (Table S2). On the other hand, if the passive method is considered as a "golden standard" then the reference value for the slope parameter should be 1. In this case, a statistically significant difference was recorded between the values ​​obtained at the seasonal level and the chosen reference value. The relative percentage difference (RPD) calculated for the two methods indicate a value higher than 25% for 17% (summer data) and 30% (winter data) of the houses, respectively.

### Temporal correction factors for IRC using two different measuring methods

**Figure 2:**
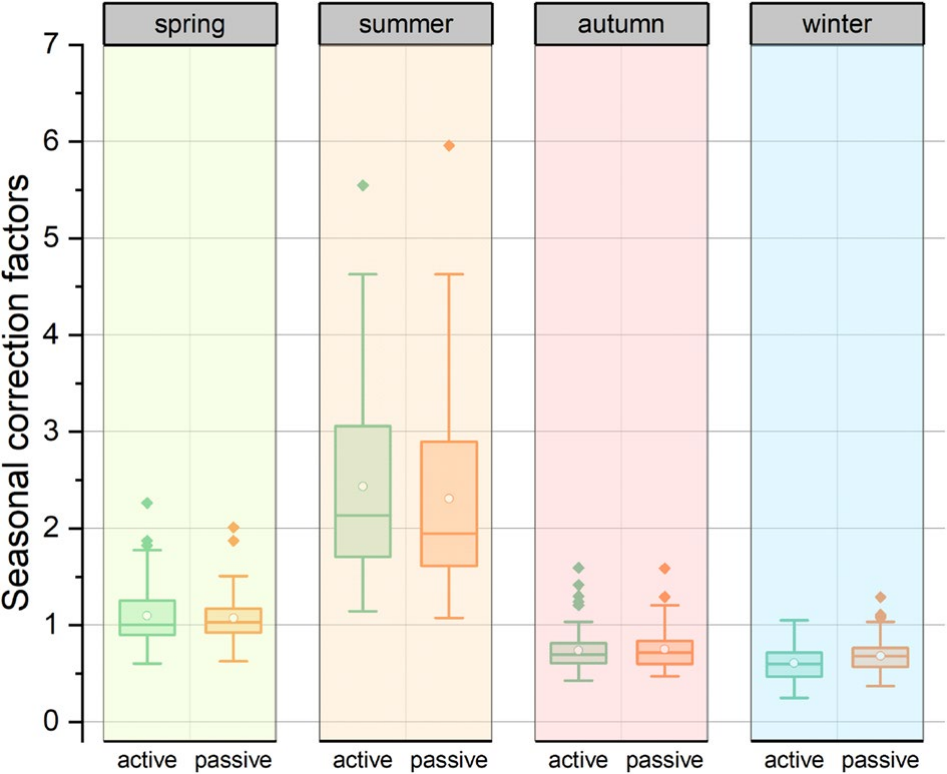
Temporal correction factors for indoor radon concentrations computed at season level for the passive and active methods. The figure was made using OriginPro 2019b software (www.originlab.com).

According to the data collected in the present study, illustrated in Fig. 2, as well as other similar studies^19–21^, seasonal correction factors obtained for the summer season for both passive and active methods show a high variability. The seasonal correction factors showed a log-normal distribution, confirmed by applying the Shapiro–Wilk test on log-transformed data. A detailed descriptive statistics of temporal correction factors depending on the measuring method is presented in Table 2. The geometric means for the TCF during the summer season are 2.26 (ranging between 1.14 and 5.55) for the passive method and 2.16 (ranging between 1.08 and 5.96) for the active method. In the case of the spring season, the geometric means are 1.06 (passive method) and 1.05 (active method). For the winter season the two methods provided closely similar results in terms of geometric means (0.59 and 0.61), while for the autumn season the geometric means obtained by the two methods are identical (0.72). A paired-samples t-test on log-transformed data was conducted to compare the temporal correction factor according to the measurement method used at season level. The differences between the TCF values for the two methods were statistically significant only for the summer season, even after removing outliers (t = 2.53, df = 67, *p* = 0.02).

**Table 2:** The descriptive statistics of seasonal correction factor for indoor radon concentrations measured by two different methods in 68 retrofit houses.

Season	Measurement method	Min	Max	A.M	S.D	G.M	G.S.D
Spring	p	0.60	2.26	1.10	0.31	1.06	1.30
	a	0.62	2.01	1.07	0.24	1.05	1.24
Summer	p	1.14	5.55	2.44	0.97	2.26	1.46
	a	1.08	5.96	2.31	0.92	2.16	1.44
Autumn	p	0.43	1.59	0.74	0.22	0.72	1.30
	a	0.47	1.59	0.75	0.22	0.72	1.31
Winter	p	0.25	1.05	0.61	0.17	0.59	1.34
	a	0.37	0.98	0.63	0.13	0.61	1.24

Three of the surveyed buildings were treated as outliers, being excluded from this statistical analysis.

p—passive measurements using CR-39 detectors; a—active measurements using ICA system with TSRS2 radon sensor; A.M.—arithmetic mean; S.D.—standard deviation; G.M.—geometric mean; G.S.D.—geometric standard deviation.

The TCF was found to vary widely from house to house, as can easily be observed in Fig. 2. Therefore, special attention was given to TCF analysis depending on the city, in order to identify variations induced by the region in which the measurements take place.

*Passive method.* A statistically significant difference was found for the spring season between the medians of TFC values for Sibiu and Cluj-Napoca, respectively Sibiu and Timişoara, by applying K-W test. On the other hand, the differences were found to be statistically significant between Timişoara and the other cities involved in the analysis, in the case of TCF associated with the winter season. The results obtained for the winter agree with the results obtained for the outdoor temperature, in Timişoara the mean during winter season (2.7 °C) being significantly higher than the average values specific for the other cities.

*Active method.* A statistically significant difference was found between the medians of TCF values during the spring season between Sibiu and Cluj-Napoca and during the summer season between Cluj-Napoca and Sibiu, respectively Cluj-Napoca and Timişoara. In this case, the outdoor temperature differences are more subtle between Cluj-Napoca and Sibiu, with an average difference of 0.5 °C, while between Cluj-Napoca and Timişoara the temperature difference is about 2 °C.

### Graphical representation of IRC measured by ICA system

**Figure 3:**
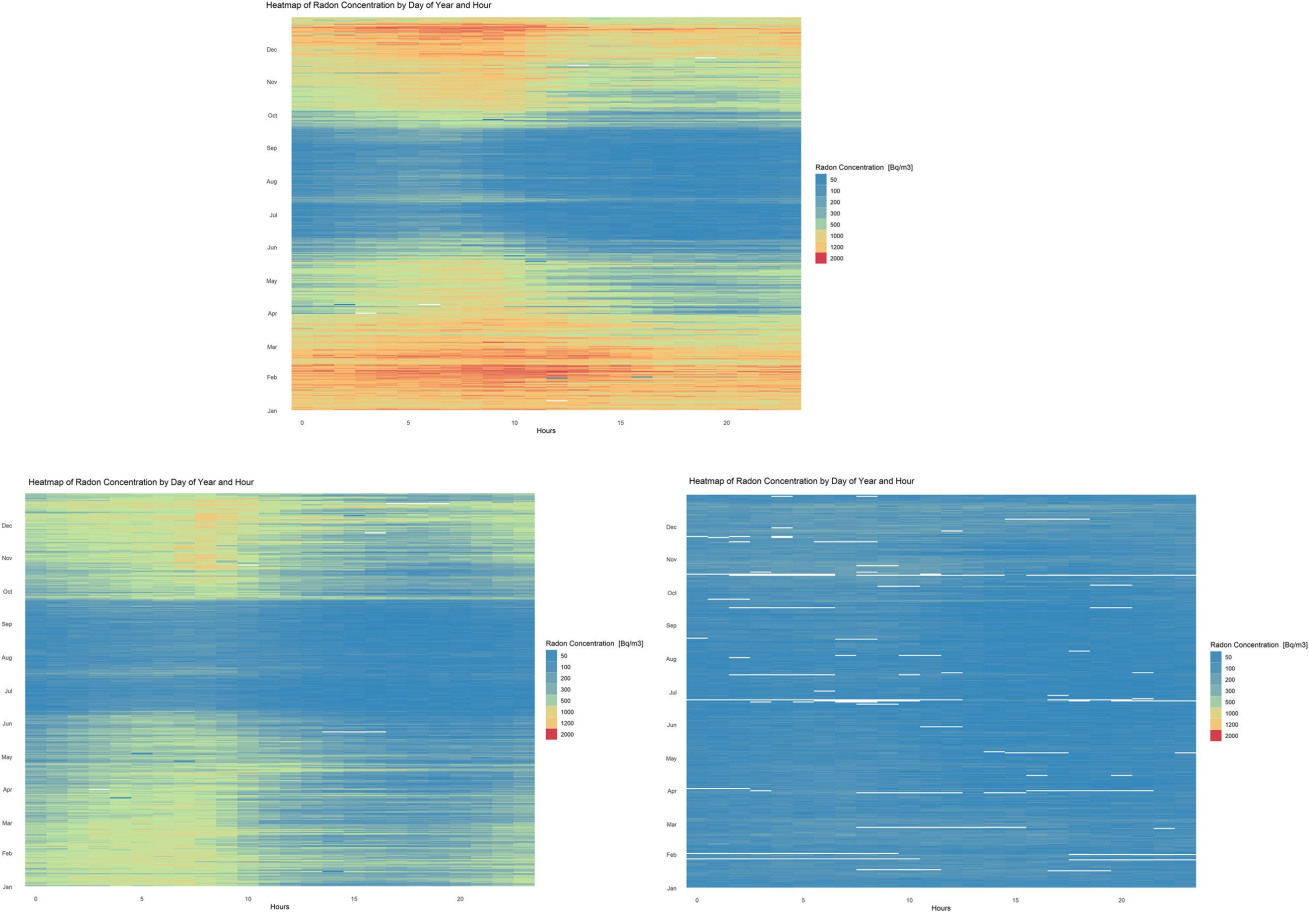
The heat map representation of radon concentration during one year. AIRC of 665 Bq/m^3^ (top), 335 Bq/m^3^ (left) and 65 Bq/m^3^ (right). Each “pixel” represents the average hourly radon conc. The white pixel represents the missing data. The figure was made using R (3.6.3) software (www.rstudio.com).

A fast and efficient technique, easy to interpret even by the homeowner, in order to identify the "hot" periods in which the concentration of the monitored parameters exceeds certain threshold values (such as 300 Bq/m^3^ for radon concentrations) ​​is the heat map representation. In Fig. 3 are represented the hourly averages of radon concentration during a day (abscissa), for the investigated period (in this case one year, with a daily resolution). In this sense, three distinct houses were chosen with annual mean of radon concentrations of 665 Bq/m^3^, 335 Bq/m^3^, and 65 Bq/m^3^, respectively, in order to highlight the importance of temporal variations.

**Figure 4:**
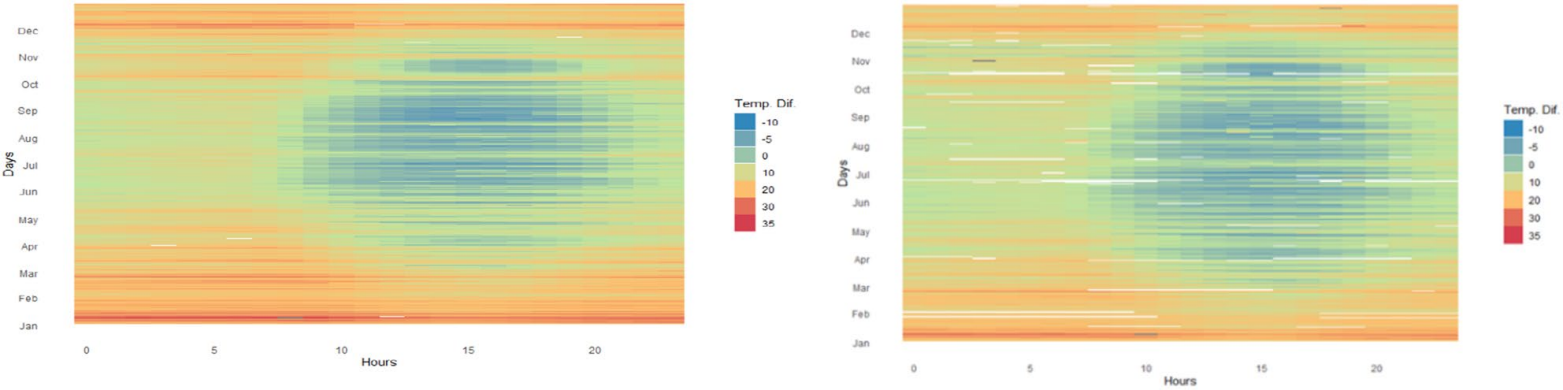
The heat map representation of Δt (tin–tout): Δt for the house with AIRC of 665 Bq/m^3^ (left), Δt for the house with AIRC of 65 Bq/m^3^ (right). Each “pixel” represents the average hourly Δt. The figure was made using R (3.6.3) software (www.rstudio.com).

In a similar manner, the temperature difference (indoor–outdoor) is displayed in Fig. 4, choosing the houses with extreme AIRCs, i.e. 665 and 65 Bq/m^3^. It has been well documented that temperature has a significant impact on variations in radon concentration^42,43^. In the present study the differences are obvious (Fig. 3 top and bottom right) in terms of radon concentration, but less evident in terms of temperature differences (Fig. 4).

**Figure 5:**
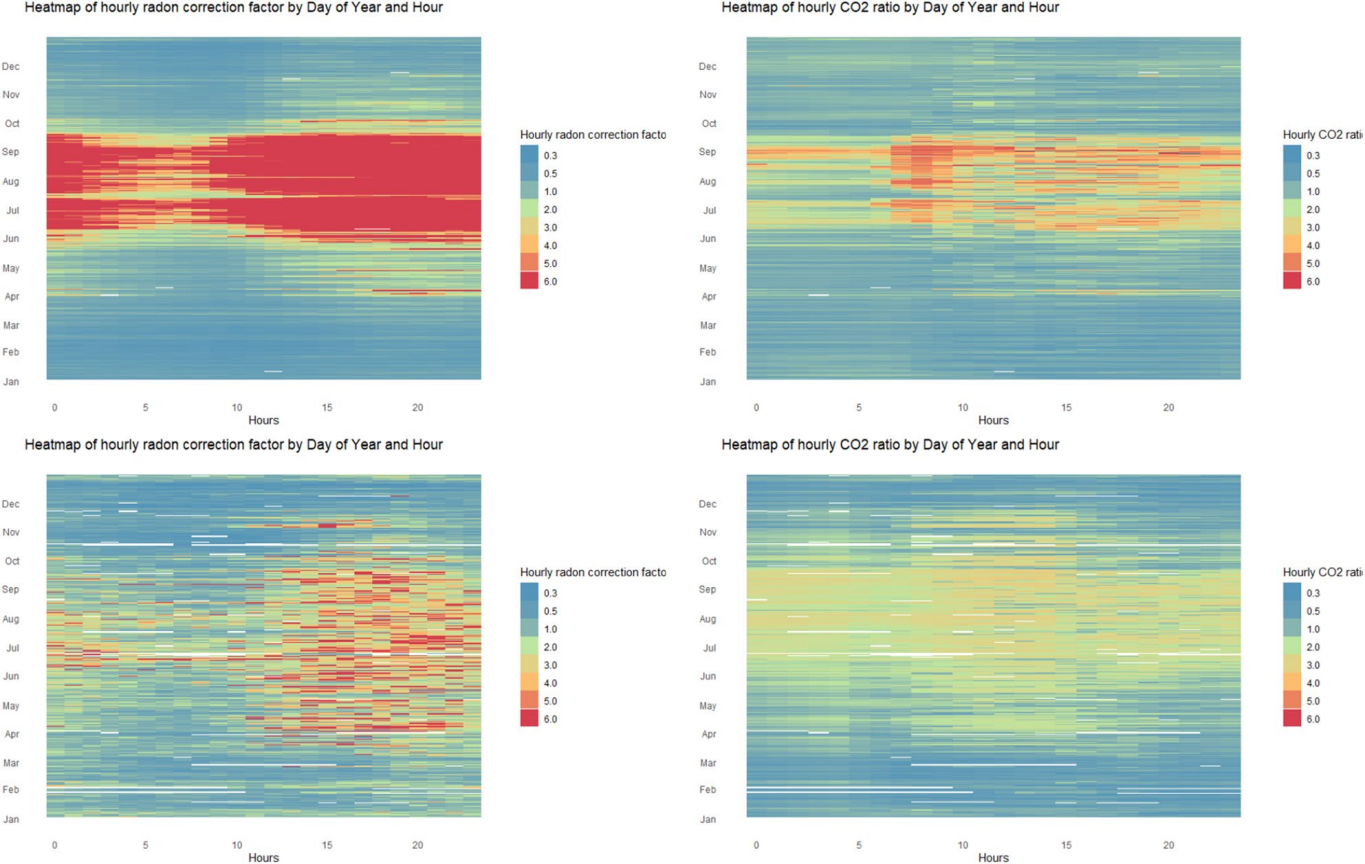
The heat map representation of TCF for radon concentration during one year (left): AIRC of 665 Bq/m^3^ (top), AIRC of 65 Bq/m^3^ (bottom). On the right are represented the ratios between the annual CO_2_ concentration and the hourly means for the two houses. The figure was made using R (3.6.3) software (www.rstudio.com).

Following this graphical representation, the most striking association was obtained between the TCF for radon concentration and the ratios between the annual and the hourly CO_2_ concentration, monitored by ICA system. The CO_2_ concentration is a good indicator for human bioeffluent emissions. Therefore, a hypothesis was formulated according to which the ratio between the annual CO_2_ concentration and the hourly means could reflect the changes related to the use of the house, i.e. intervals and rate of ventilation. Figure 5 shows the TCF for radon concentration for the house with AIRC of 665 Bq/m^3^ (top, left), respectively with 65 Bq/m^3^ (bottom, left) and the ratios of the annual CO_2_ concentration per hourly concentration corresponding to each house (Fig. 5, right, top and bottom).

### Monthly correction factors for indoor radon using ICA system

**Figure 6:**
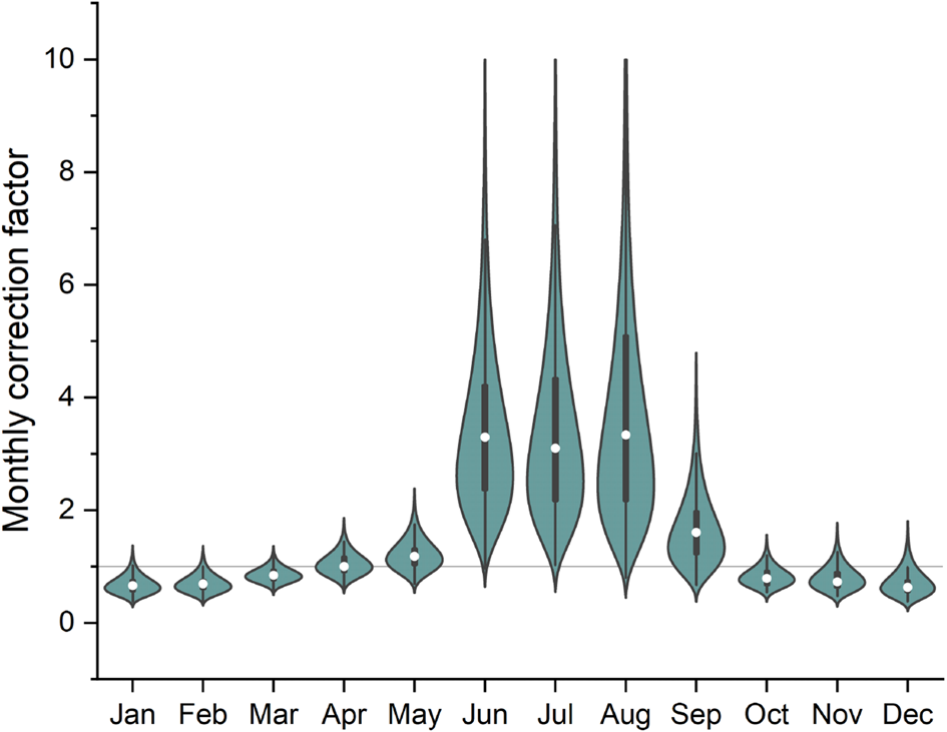
Temporal correction factors for indoor radon concentrations computed at month level using the active method. The figure was made using OriginPro 2019b software (www.originlab.com).

The data obtained for the 71 houses allowed the calculation of radon correction factors with a monthly resolution. The analysis of the obtained data indicates a lognormal distribution for the monthly correction factors. The overlap of data reported in Figs. 2, 6 and Table S3 indicate that the highest dispersion of correction factors is specific to the summer months with a maximum geometric mean of 3.23 for August (ranging between 0.80 and 9.82), while the lowest geometric mean (0.66), ranging between 0.38 and 1.12 was associated with a winter month, namely January. The monthly correction factors showed no statistically significant differences depending on the city where the measurement took place.

Table S4 shows the correction factors for radon concentration depending on the starting month and the duration of the measurement (from one month to 11 months). The usefulness of such tabulations is represented by the fact that it allows the use of a correction factor according to each combination of the starting month of the measurement and the duration of exposure. However, in order to reduce the uncertainty related to the estimation of the annual concentration, it is desired that the variability between the measurements be as low as possible, so that the average value of the correction factor to be representative.

The coefficient of variation for the TCF has been listed in Table S5. A close analysis of the results indicates that a lower value (less than 10%) assumes measurements of 7 + months, while a variability close to 20% can be reached by measurements of approx. 4 months, except for measurements starting in the summer months.

### Factors related to TCF using GEE method

As previously mentioned, the longitudinal design of the study led to the use of GEE analysis. The multivariate analysis was applied by using the monthly average values ​​(12) for the investigated factors for all 71 houses. Overall, 852 lines (12 × 71) were used in this analysis. Tabulated estimates from GEE method for the temporal correction factors, assuming an autoregressive (AR (1)) correlation structure, are presented in Table 3. The month of May was chosen as the reference category, due to the fact that it presents a geometric mean close to the geometric mean of all measurements both in terms of TCF and r_CO2_. The independent variables were included in the analysis, according to the statistical significance (*p* < 0.05).

**Table 3:** Factors related to temporal correction factors for indoor radon concentration using GEE method.

Parameter	Beta	SE	*p* value
(Intercept)	0.153	0.231	0.51
**Month**^**a**^			
[month = 1]	0.129	0.269	0.63
[month = 2]	0.079	0.234	0.74
[month = 3]	0.220	0.255	0.39
[month = 4]	0.309	0.318	0.33
[month = 5]^b^	0		
[month = 6]	− 0.604	0.606	0.32
[month = 7]	− 0.604	0.438	0.17
[month = 8]	− 0.837	0.502	0.10
[month = 9]	− 0.239	0.320	0.45
[month = 10]	0.151	0.233	0.52
[month = 11]	− 0.129	0.288	0.65
[month = 12]	0.117	0.266	0.66
**r**_**CO2**_	1.029	0.235	< 0.001
**Month** × **r**_**CO2**_			
[month = 1] × r_CO2_	− 0.521	0.296	0.08
[month = 2] × r_CO2_	− 0.470	0.248	0.06
[month = 3] × r_CO2_	− 0.476	0.261	0.07
[month = 4] × r_CO2_	− 0.414	0.334	0.22
[month = 5] × r_CO2_	0		
[month = 6] × r_CO2_	1.539	0.476	< 0.001
[month = 7] × r_CO2_	1.249	0.345	< 0.001
[month = 8] × r_CO2_	1.326	0.379	< 0.001
[month = 9] × r_CO2_	0.249	0.296	0.40
[month = 10] × r_CO2_	− 0.509	0.239	0.03
[month = 11] × r_CO2_	− 0.174	0.305	0.57
[month = 12] × r_CO2_	− 0.572	0.278	0.04

Dependent variable: Radon temporal correction factors.

^a^The reference category was month = 5.

^b^This parameter is set to zero because it is redundant.

A statistically significant effect of the month in which the measurement took place (*p* = 0.016), r_CO2_ (*p* < 0.001), as well as the interaction of the two parameters was obtained (*p* < 0.001) by applying the gamma distribution with identity link on the temporal correction factors. According to the data reported in Table 3, there is a significant association of CO_2_ ratio with radon TCF for May (the reference level of time factor), the estimated parameter being 1.029. This value suggests that in the case of measurements made in May, an increase with one unit in r_CO2_ results in an increase of 1.029 in mean of radon TCF. There is also a significant interaction between r_CO2_ and month, the slope increasing between 0.249 (September) and 1.539 (June). For the rest of the months, the slope showed a decrease between -0.174 for November to -0.572 for December. A statistically insignificant differences between intercepts were observed between the reference category and the rest of the months. Other factors, i.e. indoor temperature, temperature differences between indoor and outdoor, the relative humidity, and city ID were added, but they were ultimately removed from the analysis because their impact was not statistically significant.

## Discussions

Considering the continuous growth of interest about indoor air quality, as well as the miniaturization and cheapening of sensors, along with the many advantages of continuous, real-time monitoring, a paradigm shift related to how the radon measurements are performed is expected. The introduction of graphic representations, similar to those presented in Figs. 3, 4 and 5 can be a point of support both among specialists and among the general public to observe the existence of certain patterns over time, giving them the opportunity to make changes in behaviour in order to improve indoor air quality.

While the international organisations^37,38^ recommend the passive method as a golden standard for AIRC, a constant pressure is manifested both by the end user, who in certain situations cannot afford to allocate such a long time (months) to obtain the result, as well as from the service provider for which a shorter duration of time contributes to the increase of business and efficiency. Unfortunately, until recently, this reduction in time allotted for radon measurements was accompanied by a reduction in the accuracy of AIRC estimation. In addition, a major problem in estimating the AIRC on the basis of monthly or seasonal measurements is the use of temporal correction factors. Applying an average value for TCF specific to a particular country or region often leads to much higher uncertainties than those inherent to the measurement method. Consequently, any additional information that may reduce this uncertainty is highly desired.

The results obtained in the present study indicate that the active measurements with the new generation of continuous, online, real-time monitoring devices, can provide a credible result for the measured indoor radon concentrations. According to the season, between 12 (summer) and 21 (winter) of the 71 monitored houses showed a relative percentage difference higher than 25%, which is considered as a threshold value beyond which the recalibration of the active method is required.

The significant difference in terms of radon concentrations between the active and passive measurements represented in Fig. 1 and indicated as regression parameters in the Table S2 may suggest the need for a correction in the case of active measurements, which would adjust the measured radon concentration according to physical parameters, i.e. relative humidity and temperature. Among the factors that led to this discrepancy can be mentioned the fact that for ICA systems the calibration reference was the AlphaGuard device (PQ2000 PRO; Saphymo GmbH, Frankfurt, Germany) and not the passive method. In addition, following the international intercomparison performed by the BfS Radon Calibration Laboratory from Germany, although our results for the passive method showed a deviation below 10% from the reference value, the obtained results by our laboratory overestimated the real value. A similar situation was identified in the present study, where the values ​​provided by the passive method are generally higher than those obtained by the active method.

Despite these differences, for the TCF computed according to the two methods, a statistically significant differences were recorded only for the summer season. Furthermore, the additional measurements made in the same period of time (CO_2_ concentration, temperature, etc.) could offer supplementary details about the indoor environment, which can provide a much more accurate picture of the TCF applied for AIRC estimation. A much clearer picture on how user behaviour shapes the monthly evolution of TCF comes in the form of CO_2_ ratio, which may vary throughout the year as a result of indoor activities and environmental parameters. The main drawback of the relation between TCF and the CO_2_ ratio is given by the need to use the annual average of CO_2_ in order to calculate the last parameter.

Although, from an applicative point of view, the usefulness of this parameter is limited, from a scientific point of view it can represent a new direction of investigation on how user’s behaviour, converted by this CO_2_ ratio, can affect the variation of TCF for radon concentration over time. The data analysis indicated that, the monthly CO_2_ concentrations, obtained in the present survey during March–April, respectively October–November, can be used as an approximation of the annual concentration. As a practical example, the average concentration of CO_2_ obtained in May was used as a surrogate for the annual concentration in order to calculate the CO_2_ ratio. Subsequently, the TCF obtained experimentally with ICA device for August (the month with the highest variations of TCF within the houses) were compared with: 1) the average value used in the traditional approach for the measured period, and 2) the calculated values based on the relation provided by the parameters shown in Table 3. The use of the relation between TCF and the CO_2_ ratio, led to an improvement in the estimate by reducing the RPD between 1 and 200%. Consequently, even by using the CO_2_ average for May, as a surrogate for the annual CO_2_ average, the overall improvement expressed as the mean of RPD was 20%. Although statistically significant differences were recorded between radon concentrations depending on the city in which the study was conducted, the impact of this variable was statistically insignificant in the analysis using GEE. A similar situation was recorded for the indoor-outdoor temperature differences.

## Conclusions

The use of the active method based on the new trend of indoor air quality monitoring devices, including radon, could be a viable alternative to traditional radon measurements performed by the passive method, especially for limited periods and even more during the summer season. Moreover, these devices can monitor physical parameters, such as indoor temperature, atmospheric pressure, relative humidity, as well as other indoor pollutants (CO, VOC, formaldehyde, etc.) or CO_2_. In this way, these devices can provide a much broader overview of indoor pollutants exposure. At present, we face a paradox according to which the increase of the accuracy of the estimated annual exposure it does not necessarily require a more precise and, implicitly, more expensive sensor, but rather by providing complementary information to help understand the specific dynamics in each building. Although radon measurements may not be as accurate as those offered by a professional and much more expensive active devices, by simultaneous monitoring of several parameters, indoor air quality monitoring devices can provide a much more accurate estimation of the annual exposure compared to long-term, passive radon monitoring.

This study is one of the first international attempts to monitor by active method a large number of houses for a long period of time, as a result of the decline in costs, respectively to the increase in accuracy of the sensors used. The results obtained can provide the missing tool, i.e. tabulated temporal correction factors, in assessing annual radon concentration based on measurements performed for shorter periods of time, using the active method. Moreover, the association between the radon TCF and the CO_2_ ratio can lead to an increase in the accuracy with which AIRC is estimated. The survey represents a premiere in assessing temporal correction factors for indoor radon, on a large-scale, undertaken in Romania. Therefore, we consider that this study represents a bridge between the traditional method of measuring the radon concentration—the passive method—and the monitoring devices of the environmental parameters available to the general public. In addition, the study could provide the missing link in aligning with the global trend of using smart devices, with results provided in real time, thus attracting the public’s interest in radon measurements.

## Materials and methods

### Study area and building selection

Considering the general trend of working from home, which leads to an increase in the occupancy factor inside the buildings, as well as that of reducing energy consumption by the thermal retrofit of the buildings, 100 energy efficient residential buildings, from five Romanian cities (Bucharest, Cluj-Napoca, Iaşi, Sibiu and Timişoara), were selected for the present study.

**Figure 7:**
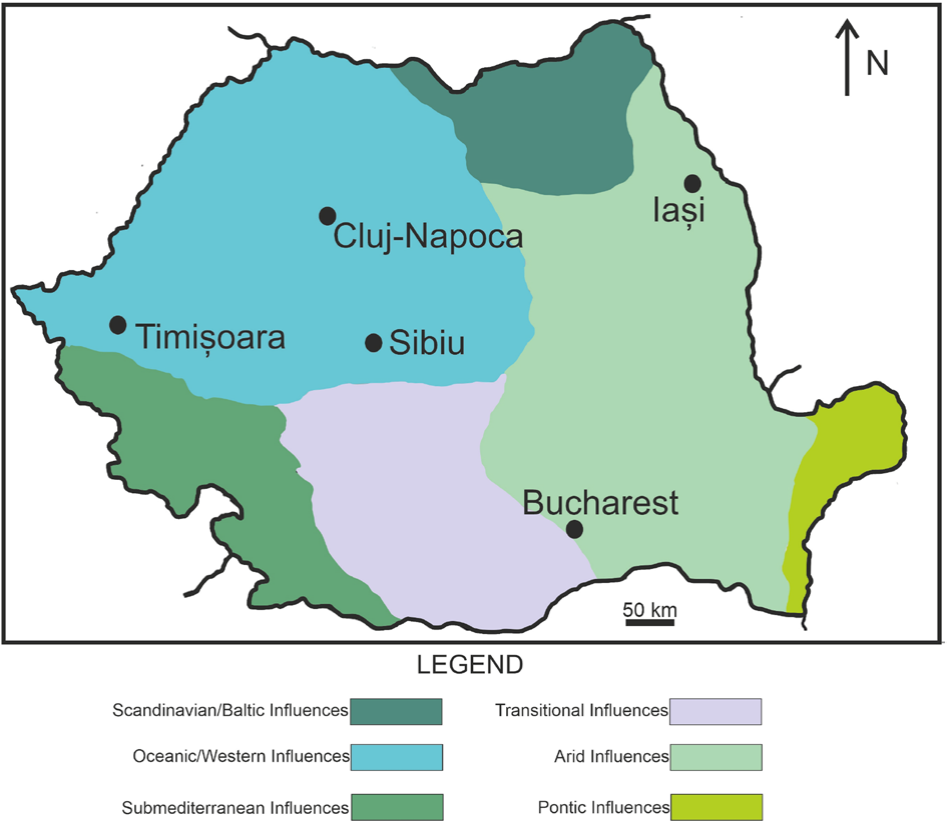
The cities involved in the analysis and the climatic influences in Romania. The map was made in the CorelDraw 2020 software (www.coreldraw.com).

All five cities overlap with the temperate continental climate zone, characterized by a significant climatic contrast between the hot and cold seasons, which is specific to the whole area of South-Eastern Europe^44^. Due to the geographical position, the cities of Timişoara, Sibiu and Cluj-Napoca have a moderate temperate continental climate, characterized by a regime of quantitatively significant rainfall, with significantly reduced droughts (Fig. 7). The predominantly western and north-western air circulation, with important maritime influences, will make the annual average air temperature for the three mentioned cities slightly lower than in the case of Iaşi and Bucharest^44–46^. On the other hand, the cities of Bucharest and Iaşi are characterized by a more pronounced continentalization, with tendencies of excess. This translates into a relatively moderate rainfall regime, but which occurs at irregular intervals and in different amounts, usually preceded by long periods of drought.

The residential buildings were selected applying the following criteria: (1) previous measurements that indicated an AIRC higher than 250 Bq/m^3^; (2) the presence of thermal insulation of the walls or an upgrade of the windows. Written informed consent was obtained from all participants for involving their house in the study, analysis and publication of results.

### Passive radon measurements and analysis

The indoor radon measurements were performed using CR-39 track detectors (Radosys Ltd. Hungary). In order to measure the indoor radon concentration associated with different seasons, the detectors were placed in four successive campaigns of 3 months each. The corresponding months for the four Romanian seasons are as follows: winter—December to February, spring—March to May, summer—June to August and autumn—September to November. Thus, the AIRC was computed as geometric mean of the four seasonal measurements. The detectors were installed by a member of the research team; the targeted room was the bedroom, but in certain situation, the living room was the only room provided by the owners. After each exposure period, the detectors were collected by the research team or sent by courier to the laboratory, where they were processed and analysed according to the LiRaCC laboratory protocol described by Cucoş et al.^47^. The same batch of detectors were used for the entire experiment in order to avoid uncertainties do to manufacturing issues. The performance in intercomparison and proficiency testing for passive radon measurements, carried out yearly at the BfS Radon Calibration Laboratory in Germany, provided below 10% deviation from reference value, respectively, satisfactory results^48^.

### Active measurements

Active devices, named ICA system, developed by the members of the LiRaCC, were installed in each of the 100 houses, in the same room where the passive detector was placed. The ICA system was developed with the TSRS2 radon sensor (Tesla, Czech Rep.). Additionally, the ICA system incorporates sensors for continuous, real-time monitoring of CO_2_, CO and VOC concentrations, as well as indoor ambient conditions, such as temperature, pressure and relative humidity (RH). The calibration of the sensors was performed prior to installation using a reference device traceable to PTB primary radon standard. Beldean-Galea et al.^31^ present the main characteristics of the sensors from the ICA system. Due to various reasons (misplacement, loss, etc.), the passive detectors linked to all seasonal exposures were retrieved from only 71 houses. The data recorded continuously by the ICA system in these same houses did not show losses higher than 5%. The following analyses will, therefore, refer to the data obtained for these 71 houses.

The data ​​of the external physical factors (temperature, relative humidity and atmospheric pressure) were obtained from the local meteorological stations, being specific at city level and not for each individual house.

In order to differentiate the results obtained by the two measurement methods, the values specific to the passive method will be denoted with index p, and those for the active method with index a.

### Temporal correction factors for indoor radon concentration

The TCF for indoor radon concentration measured with passive detectors were computed as a ratio between the annual mean and the means obtained for each season. Although ICA transmits data every 2 min, due to the enormous volume of data, an hourly mean was computed.

In the case of radon measurements, the TSRS2 sensor performs a moving average for the last 15 measurements just to avoid the volatility of short-term measurements. Possible missing data, caused by interruptions in the ICA's functionality (i.e. power supply and/or internet connection issues) were replaced as an average of the neighbouring values. Subsequently, starting from these hourly means, the means for each month, respectively the AIRC mean were calculated. The geometric mean was preferred in order to minimize the impact of outliers, respectively due to the lognormal distribution of the data regardless of the time resolution. The ratio of the annual and the monthly means of IRC allowed the calculation of the seasonal correction factors with a monthly resolution. Considering the continuity of data flow over time, offered by ICA, a table containing the TCF for any combination between the starting month of the measurement and the number of months as exposure period was possible.

### Statistical analysis

The statistical analysis of the data was performed using IBM SPSS 24 (IBM Corp., Armonk, NY, USA) and OriginPro 2019b (OriginLab Corporation, Northampton, MA, USA) software. The Shapiro–Wilk test was used to verify the normality of data distribution. The t-test was performed for two paired samples, while Mann–Whitney test (M-W test) was applied for two independent samples. Kruskal–Wallis test (K-W test) with Dunn’s post-hoc analysis for more than two samples was used for unpaired comparisons. In the case of more than two paired samples, ANOVA for repeated measures was chosen. The intensity of the relation between the examined variables was calculated using Pearson correlation coefficient. The degree of agreement between the two methods was evaluated by Lin’s concordance correlation coefficient. The significance level α was chosen at 0.05. Due to the log-normal distribution, in some situations the data were summarized by GM.

The generalized estimating equations (GEE) was used for the longitudinal data analysis. The dependent variable is TCF obtained by active measurements and represented as a monthly average. The independent variables were represented by the ratios between annual CO_2_ concentration and monthly means (r_CO2_), the differences between the indoor–outdoor temperature (Δt) and the city ID. The variable for the month in which the determination took place was introduced as a factor in this analysis. The subjects in the model were entered by the specific ID of each house, while the within-subject variable was represented by the categorical variable of the month in which the measurement took place. The model with gamma distribution and the identity link was applied on the data of the dependent variable.

The quasi-likelihood information criterion (QIC) was used in order to choose the working correlation structure. As a general rule, a lower value for QIC indicates a better model. The autoregressive (AR (1)) provided the lower QIC between the tested correlation structure (AR (1), Exchangeable and Unstructured). The AR(1) correlation structure assumes that the measurements closer in time have a higher correlation than those that are further apart^41^.

The relative percentage difference (RPD) is generally used in radon intercomparison exercises in order to quantify the difference between the individual result and the reference value^49^. In the present study, the RPD was used to quantify the difference between the measurement obtained by active method (m_a_) and the golden standard—passive method (m_p_), using the following equation:

$$RPD\left(\%\right)=100\times \frac{\left|{m}_{a}-{m}_{p}\right|}{{m}_{p}}$$

Figures 1, 2 and 6 were made in the OriginPro 2019b, while Figs. 3, 4 and 5 in the R studio (4.0.2), using the gplots and ggplot2 packages. Figure 7 contains an adaptation of the climate map for Romania (www.geotutorials.ro) with the rendering of the position of the cities involved in the study. The map was made using CorelDraw 2020 software.

https://www.coreldraw.com/

www.geotutorials.ro/atlas-geografic/harti-romania/atlas-geografic-1980/harta-climatica-romania/

www.originlab.com

www.rstudio.com

## Supplementary Information

Supplementary Information
