# Supplementary material for: A new approach to radon temporal correction factor based on active environmental monitoring devices
Source: Sci Rep. 2021 May 11;11:9925. doi: 10.1038/s41598-021-88904-2 (PMC8113422; doi:10.1038/s41598-021-88904-2)
Supplement: Supplementary file 1 — Supplementary Information [file 41598_2021_88904_MOESM1_ESM.docx]

***Table S1.*** *The descriptive statistics of indoor radon concentration (Bq/m^3^) depending on the city and the season for passive measurements*

| *Season* | City | N | A.M. | S.D. | Med. | Minimum | Maximum | G.M. |
| --- | --- | --- | --- | --- | --- | --- | --- | --- |
| *Spring* | Bucharest | 8 | 286 | 160 | 237 | 82 | 588 | 247 |
|  | Cluj-Napoca | 33 | 350 | 166 | 366 | 80 | 895 | 311 |
|  | Iaşi | 5 | 242 | 73 | 250 | 153 | 331 | 232 |
|  | Sibiu | 8 | 159 | 70 | 148 | 64 | 261 | 144 |
|  | Timişoara | 17 | 371 | 134 | 375 | 178 | 711 | 349 |
| *Summer* | Bucharest | 8 | 124 | 48 | 116 | 66 | 213 | 116 |
|  | Cluj-Napoca | 33 | 121 | 66 | 110 | 34 | 277 | 105 |
|  | Iaşi | 5 | 106 | 47 | 98 | 57 | 168 | 97 |
|  | Sibiu | 8 | 146 | 58 | 123 | 84 | 264 | 138 |
|  | Timişoara | 17 | 211 | 80 | 175 | 112 | 427 | 199 |
| *Autumn* | Bucharest | 8 | 489 | 234 | 547 | 152 | 776 | 426 |
|  | Cluj-Napoca | 33 | 485 | 221 | 434 | 177 | 1108 | 442 |
|  | Iaşi | 5 | 466 | 59 | 494 | 399 | 525 | 463 |
|  | Sibiu | 8 | 374 | 145 | 352 | 228 | 687 | 353 |
|  | Timişoara | 17 | 457 | 169 | 446 | 236 | 924 | 430 |
| *Winter* | Bucharest | 8 | 726 | 457 | 613 | 240 | 1654 | 613 |
|  | Cluj-Napoca | 33 | 672 | 373 | 574 | 272 | 1469 | 585 |
|  | Iaşi | 5 | 661 | 312 | 583 | 354 | 1187 | 611 |
|  | Sibiu | 8 | 372 | 138 | 366 | 197 | 575 | 348 |
|  | Timişoara | 17 | 483 | 195 | 457 | 218 | 1047 | 452 |
|  | Bucharest | 8 | 320 | 137 | 284 | 146 | 539 | 294 |
|  | Cluj-Napoca | 33 | 309 | 111 | 309 | 136 | 533 | 289 |
| *Annual* | Iaşi | 5 | 287 | 54 | 294 | 210 | 355 | 283 |
|  | Sibiu | 8 | 230 | 61 | 230 | 131 | 320 | 222 |
|  | Timişoara | 17 | 353 | 96 | 353 | 191 | 589 | 341 |

N – number of houses; A.M. – arithmetic mean; S.D. – standard deviation; Med. – Median; G.M. – geometric mean.

***Table S2.*** *The regression analysis for the two measuring methods of indoor radon concentrations at the season level*

| Season | Parameter | Value | S.E. | t-value | p-value |
| --- | --- | --- | --- | --- | --- |
| Spring | Intercept | 29.939 | 16.490 | 1.816 | 0.074 |
|  | Slope | 0.813 | 0.048 | 16.936 | **<0.001** |
| Summer | Intercept | 12.461 | 7.557 | 1.648 | 0.104 |
|  | Slope | 0.862 | 0.048 | 17.870 | **<0.001** |
| Autumn | Intercept | 20.877 | 27.959 | 0.747 | 0.458 |
|  | Slope | 0.862 | 0.055 | 15.450 | **<0.001** |
| Winter | Intercept | 141.807 | 31.808 | 4.458 | **<0.001** |
|  | Slope | 0.592 | 0.048 | 12.451 | **<0.001** |
| Annual | Intercept | 7.385 | 12.669 | 0.583 | 0.561 |
|  | Slope | 0.884 | 0.038 | 23.485 | **<0.001** |

***Table S3.*** *The descriptive statistics of monthly correction factor for indoor radon concentration using ICA system*

| Month | N* | A.M. | S.D. | G.M. | G.S.D. | Min. | Median | Max. |
| --- | --- | --- | --- | --- | --- | --- | --- | --- |
| January | 71 | 0.68 | 0.17 | 0.66 | 1.28 | 0.38 | 0.66 | 1.12 |
| February | 71 | 0.70 | 0.17 | 0.68 | 1.26 | 0.38 | 0.69 | 1.38 |
| March | 71 | 0.85 | 0.13 | 0.84 | 1.17 | 0.57 | 0.85 | 1.18 |
| April | 71 | 1.04 | 0.21 | 1.02 | 1.22 | 0.60 | 1.00 | 1.62 |
| May | 71 | 1.21 | 0.30 | 1.18 | 1.26 | 0.69 | 1.18 | 2.30 |
| June | 69 | 3.44 | 1.47 | 3.15 | 1.54 | 0.96 | 3.30 | 8.38 |
| July | 69 | 3.47 | 1.63 | 3.13 | 1.60 | 1.02 | 3.10 | 8.00 |
| August | 68 | 3.68 | 1.90 | 3.23 | 1.69 | 0.80 | 3.34 | 9.82 |
| September | 71 | 1.68 | 0.70 | 1.56 | 1.48 | 0.67 | 1.61 | 4.31 |
| October | 71 | 0.82 | 0.19 | 0.80 | 1.25 | 0.54 | 0.79 | 1.43 |
| November | 71 | 0.81 | 0.25 | 0.77 | 1.32 | 0.47 | 0.73 | 1.63 |
| December | 69 | 0.73 | 0.31 | 0.68 | 1.40 | 0.38 | 0.63 | 1.92 |

*Some outliers were excluded from analysis.

***Table S4.*** *The geometric mean of radon temporal correction factor using ICA system in 71 houses depending on the duration of the measurement*

| Month | 1 | 2 | 3 | 4 | 5 | 6 | 7 | 8 | 9 | 10 | 11 |
| --- | --- | --- | --- | --- | --- | --- | --- | --- | --- | --- | --- |
| January | 0.66 | 0.65 | 0.70 | 0.76 | 0.81 | 0.92 | 1.02 | 1.11 | 1.14 | 1.09 | 1.05 |
| February | 0.68 | 0.73 | 0.81 | 0.87 | 1.01 | 1.13 | 1.24 | 1.26 | 1.18 | 1.12 | 1.05 |
| March | 0.84 | 0.91 | 0.97 | 1.17 | 1.32 | 1.46 | 1.45 | 1.31 | 1.21 | 1.12 | 1.05 |
| April | 1.02 | 1.06 | 1.35 | 1.55 | 1.72 | 1.66 | 1.43 | 1.28 | 1.16 | 1.08 | 1.02 |
| May | 1.18 | 1.63 | 1.91 | 2.09 | 1.91 | 1.54 | 1.34 | 1.19 | 1.09 | 1.02 | 1.00 |
| June | 3.44 | 2.91 | 2.92 | 2.32 | 1.65 | 1.38 | 1.20 | 1.08 | 1.01 | 0.99 | 0.99 |
| July | 3.47 | 2.90 | 2.16 | 1.49 | 1.24 | 1.09 | 0.99 | 0.93 | 0.92 | 0.93 | 0.94 |
| August | 3.68 | 1.92 | 1.28 | 1.09 | 0.97 | 0.90 | 0.85 | 0.85 | 0.86 | 0.88 | 0.94 |
| September | 1.56 | 1.00 | 0.90 | 0.83 | 0.79 | 0.76 | 0.77 | 0.79 | 0.82 | 0.88 | 0.94 |
| October | 0.80 | 0.76 | 0.73 | 0.71 | 0.70 | 0.72 | 0.75 | 0.78 | 0.85 | 0.91 | 0.97 |
| November | 0.77 | 0.71 | 0.69 | 0.68 | 0.71 | 0.74 | 0.78 | 0.86 | 0.93 | 1.00 | 1.03 |
| December | 0.73 | 0.66 | 0.66 | 0.69 | 0.74 | 0.79 | 0.88 | 0.96 | 1.04 | 1.07 | 1.03 |

***Table S5.*** *The coefficient of variation (%) for radon temporal correction factor using ICA system in 71 houses*

| Month | 1 | 2 | 3 | 4 | 5 | 6 | 7 | 8 | 9 | 10 | 11 |
| --- | --- | --- | --- | --- | --- | --- | --- | --- | --- | --- | --- |
| January | 37% | 37% | 29% | 25% | 21% | 17% | 14% | 12% | 11% | 10% | 8% |
| February | 30% | 30% | 23% | 18% | 15% | 13% | 11% | 11% | 11% | 10% | 7% |
| March | 19% | 19% | 16% | 15% | 14% | 13% | 14% | 16% | 15% | 12% | 9% |
| April | 21% | 21% | 19% | 19% | 19% | 20% | 22% | 19% | 15% | 12% | 5% |
| May | 25% | 25% | 25% | 26% | 28% | 29% | 23% | 17% | 13% | 6% | 3% |
| June | 43% | 43% | 41% | 42% | 38% | 26% | 18% | 15% | 8% | 5% | 4% |
| July | 46% | 46% | 45% | 40% | 26% | 17% | 15% | 8% | 5% | 4% | 3% |
| August | 50% | 50% | 39% | 25% | 18% | 16% | 9% | 6% | 5% | 4% | 3% |
| September | 43% | 43% | 27% | 19% | 18% | 10% | 8% | 8% | 6% | 5% | 3% |
| October | 29% | 29% | 22% | 23% | 14% | 13% | 11% | 10% | 8% | 6% | 4% |
| November | 34% | 34% | 39% | 21% | 18% | 16% | 13% | 11% | 9% | 7% | 5% |
| December | 44% | 44% | 23% | 20% | 18% | 14% | 12% | 10% | 8% | 7% | 6% |

Range:

| 0 – 10 % |
| --- |
| 11 – 20 % |
| 21 – 30% |
| > 30 % |
